# Reliability and Validity of the Diabetes Eating Problem Survey in Turkish Children and Adolescents with Type 1 Diabetes Mellitus

**DOI:** 10.4274/jcrpe.4219

**Published:** 2017-12-15

**Authors:** Yasemin Atik Altınok, Suriye Özgür, Reci Meseri, Samim Özen, Şükran Darcan, Damla Gökşen

**Affiliations:** 1 Ege University Faculty of Medicine, Department of Pediatric Endocrinology, İzmir, Turkey; 2 Ege University Faculty of Medicine, Department of Biostatistics and Medical Informatics, İzmir, Turkey; 3 Ege University Faculty of Health Sciences, Department of Nutrition and Dietetics, İzmir, Turkey

**Keywords:** Diabetes eating problem survey-revised, distributed eating behaviors, type 1 diabetes mellitus, children and adolescent

## Abstract

**Objective:**

The aim of this study was to show the reliability and validity of a Turkish version of Diabetes Eating Problem Survey-Revised (DEPS-R) in children and adolescents with type 1 diabetes mellitus.

**Methods:**

A total of 200 children and adolescents with type 1 diabetes, ages 9-18 years, completed the DEPS-R Turkish version. In addition to tests of validity, confirmatory factor analysis was conducted to investigate the factor structure of the 16-item Turkish version of DEPS-R.

**Results:**

The Turkish version of DEPS-R demonstrated satisfactory Cronbach’s ∝ (0.847) and was significantly correlated with age (r=0.194; p<0.01), hemoglobin A1c levels (r=0.303; p<0.01), and body mass index-standard deviation score (r=0.412; p<0.01) indicating criterion validity. Median DEPS-R scores of Turkish version for the total samples, females, and males were 11.0, 11.5, and 10.5, respectively.

**Conclusion:**

Disturbed eating behaviors and insulin restriction were associated with poor metabolic control. A short, self-administered diabetes-specific screening tool for disordered eating behavior can be used routinely in the clinical care of adolescents with type 1 diabetes. The Turkish version of DEPS-R is a valid screening tool for disordered eating behaviors in type 1 diabetes and it is potentially important to early detect disordered eating behaviors.

**What is already known on this topic?**

Noticing and treating eating disorders in children and adolescents with type 1 diabetes is important because of their potentially severe consequences. Using a screening tool designed specifically for individuals with type 1 diabetes when assessing disturbed eating behaviors in this population is important.

**What this study adds?**

No validated disease-specific short screening tool for children and adolescents with type 1 diabetes in Turkey has so far been established. The Turkish version of Diabetes Eating Problem Survey-Revised can be used as a valid screening tool for disordered eating behaviors in type 1 diabetes. This short, self-administered diabetes-specific screening tool for disordered eating behavior can be used in the clinical care of children and adolescents with type 1 diabetes.

## INTRODUCTION

Adolescence is a developmental period with characteristic properties such as physiological changes (including weight gain, increase of adipose tissue) and psychological changes. These changes may lead to body image dissatisfaction and to an increase in prevalence of eating disorders, especially in females (1). The term “disturbed eating behaviors (DEB)”, encompasses mild as well as more extreme dieting behavior, binge eating attacks, and compensatory behavior for weight control. Studies indicate that type 1 diabetes mellitus (T1D) is a risk factor for the development of DEB (2,3). Although most of the studies focus on females, some researchers suggest that adolescent males with T1D also may have an increased risk of development of DEB (4).

Etiology of DEB is complex and multifactorial (5,6). Individual, familial, and sociocultural factors can contribute to the development of DEB (5). A lot of mechanisms have been proposed to explain the relationship between DEB and T1D. The mechanisms proossed are the effects of a chronic medical condition on body image satisfaction and low self-esteem as well as the effects of treatment of hypoglycemia and insulin use, given the focus on diet and carbohydrate intake and weight gain associated with these treatment modules (7,8). Additionally, omitting or giving less insulin than required (i.e. insulin purging) is a unique tool to reduce weight in T1D.

Early detection and treatment of eating disorders in children and adolescents with T1D is important because of their potentially severe consequences. Early detection is critical in this population in order to maintain optimum health status and decrease the chances of complications such as retinopathy, neuropathy, and diabetic ketoacidosis. The complexity of diabetes management in combination with eating disorder treatment, it is necessary to detect those most at risk as early as possible. A number of screening questionnaires and structured clinical interviews help to identify and diagnose eating disorders in children and young people with diabetes (9). It is important to use a screening measure designed specifically for individuals with T1D when establishing DEB in this population. To date, no validated disease-specific short screening tool for children and adolescents with T1D is in use in Turkey.

The Diabetes Eating Problem Survey-Revised (DEPS-R) developed by Markowitz et al (10) is a diabetes-specific self-report instrument to screen eating disorders for individuals with T1D. This present study aims to establish the reliability and validity of a Turkish version of DEPS-R in a representative sample of Turkish children and adolescents with T1D.

## METHODS

In this cross-sectional study, the children and adolescents were asked to answer a questionnaire during a regularly scheduled medical visit after written informed consent had been obtained from the subjects and their parents. All subjects were T1D patients who were being treated with multiple daily insulin injections or with insulin pump therapy. Patient records were reviewed for the following eligibility criteria:

- Duration of type 1 diabetes ≥1 year,

- A regular follow-up for at least 1 year,

- No major medical problems (celiac disease, cystic fibrosis, psychiatric disorders, or communication difficulties).

Two hundred adolescents with T1D-90 (45%) males, 110 (55%) females-aged 9–18 years were included in the study.

DEPS-R is a 16-item diabetes-specific self-report questionnaire to test for diabetes-specific eating disorders. Answers are scored on a six-point Likert scale, with higher scores indicating more DEB and a total score of ≥20 indicating a high risk for eating disorders (range 0-80). The original DEPS-R has been shown to have a good internal consistency (Cronbach’s alpha=0.86) and construct validity in a sample of pediatric population with T1D (10). Back-translation techniques were employed to develop Turkish versions of the DEPS-R. The translation techniques followed standardized procedure suggested by Brislin (11) in which the inventory items and scale were translated from English into Turkish by a professional translator.

This study was approved by the Ege University Faculty of Medicine Clinical Research Ethics Committee (approval number: 14-12.1/9). The purpose of the study was explained to each participant and written informed consent was obtained. The study procedures were in accordance with the Declaration of Helsinki.

### All subjects were asked to complete the DEPS-R.

In all subjects, height was measured to the nearest centimeter using a rigid stadiometer. Weight was measured unclothed to the nearest 0.1 kg using a calibrated balance scale. Body mass index (BMI) was calculated by the weight (kg)/height (m²) equation. Standard deviation scores (SDS) for weight, height, and BMI were calculated according to age and gender using reference values for Turkish children and the participants were categorized into four different groups as underweight (BMI-SDS <-1), of normal weight (BMI-SDS ≥-1 - <+1), overweight (BMI-SDS ≥+1 - +2< BMI-SDS), and obese (BMI-SDS ≥+2) (12).

Hemoglobin A1c (HbA1c) measurements were performed with capillary method using the NycoCard II Reader (Axis-Shield Diagnostics Ltd, Dundee, UK) device.

### Statistical Analysis

All data are presented as median for skewed data (age, HbA1c (%), duration of T1D, and DEPS-R score), mean ± SD for BMI-SDS, or percent as indicated. Normal distribution was tested for continuous data. Group differences were investigated using the t-test for normally distributed data and the Mann-Whitney U test for data not showing a normal distribution. Correlations using Pearson’s and Spearman’s correlation coefficients were calculated to explore relationships of DEPS-R score with age, HbA1c (%), duration of T1D, and BMI-SDS. Correlation values of 0.10-0.29 were interpreted as small, 0.30-0.49 as medium, and 0.50-1.0 as large (13). The factorial structure of the Turkish version of DEPS-R was examined by confirmatory factor analysis and the internal consistency was tested using Cronbach’s coefficient. Chi square tests were used for categorical variables. Level of significance was defined as p<0.05. Statistical analyses were conducted using Statistical Package for the Social Sciences (IBM SPSS Statistics) version 22.0 (SPSS Inc., Chicago, Illinois, USA) and SPSS Amos.

## RESULTS

Table 1 shows the sample characteristics. Median age of the 200 patients was 14.0 years (9.0-18.0). Median diabetes duration, HbA1c, and mean BMI-SDS values were 64.5 months (12-210), 8.05% (5.5-15.0), and 0.64±1.24 SD, respectively. There were no significant differences in age, diabetes duration, HbA1c (%) levels, and BMI-SDS values between females and males (Table 1). Seventy-one percent of the patients were on multiple daily injections (MDI) (≥4 daily injections), while 28.5 % were on insulin pump therapy. The median total daily insulin dose was 0.83 (0.46-2.09) U/for patients on MDI and 0.93 (0.23-1.53) U/kg for patients on insulin pump therapy. There was no significant difference in total daily insulin dose between MDI and insulin pump therapy groups.

**Internal consistency:** The Cronbach coefficients for the DEPS-R Turkish version were 0.847, 0.857, and 0.830 for the entire sample, females, and males, respectively

**Confirmatory factor analysis:** After the suitability of data for factor analysis was assessed, confirmatory factor analysis was performed on the 16 items of the DEPS-R Turkish version. Among the main factors and sub-factors, the established model was statistically significant (Table 2).

**Prevalence of disturbed eating behaviors risk:** The median scores obtained with the Turkish version of DEPS-R for the total sample, for females, and males were 11.0 (0-55), 11.5 (0-55), and 10.5 (0-55), respectively. There was no significant difference between females and males (p=0.122). The median scores of the DEPS-R for the MDI group was 11.0 (0-47) and 11.0 (0-55) for the insulin pump group. There was no significant difference between MDI and insulin pump therapy groups (p=0.813). A recommended cut-off score of ≥20 has been empirically established as a threshold indicating the need for further clinical assessment of eating pathology (10). A total of 29.1% of the females and 17.8% of the males scored above this cut-off value on the DEPS-R Turkish version and there was no significant difference between females and males.

Among females, the prevalence of DEPS-R scores increased steadily and significantly from 7 among underweight patients to 10 for normal weight, 21 for overweight, and 26.5 for the obese patients. Among males, the median score was 6 for underweight, 12 for normal weight and 16/15 for overweight/obese patients. There was no significant difference between overweight and obese groups in both females and males (Table 3).

### Correlations with Diabetes Eating Problem Survey-Revised Turkish Version

There were significant positive correlations of DEPS-R Turkish version scores with age (r=0.194; p<0.01), HbA1c levels (r=0.343; p<0.01), and BMI-SDS (r=0.455; p<0.01) among females. There was a positive small correlation between DEPS-R Turkish version scores and HbA1c levels (r=0.258; p<0.05) and there was positive medium correlation with BMI-SDS (r=0.351; p<0.01) among males (Table 4).

## DISCUSSION

This study examined the psychometric properties of the Turkish version of DEPS-R and its clinical utility. The DEPS-R is a 16-item instrument designed to screen for DEB in T1D. Markowitz et al (10) first used the English version of DEPS-R and showed that this screening tool had good internal consistency (Cronbach’s ∝=0.86) and validity in a representative sample of young people with T1D. As the original version, Norwegian (Cronbach’s ∝=0.89) and German versions (Cronbach’s ∝=0.84) of DEPS-R also showed that DEPS-R has a good internal consistency and validity (14,15). The Turkish version of DEPS-R showed a very good internal consistency in children and adolescents with T1D with a Cronbach’s ∝ of 0.847 (in females Cronbach’s ∝=0.85, in males Cronbach’s ∝=0.83) consistent with the previous validation studies.

The confirmatory factor analysis is a kind of structural equation model which is used to determine the relationship between observable and latent variables and has a significant value in scale adaptation studies. After the suitability of data for factor analysis was assessed, confirmatory factor analysis was performed and confirmatory factor loadings of the subscales were shown to be at an acceptable level [goodness of fit index (GFI) 0.90, adjusted GFI 0.855, comparative fit index 0.91, k2/degrees of freedom 1.82, and root mean square error of approximation 0.064].

In this sample, DEPS-R scores were similar to those reported in previous studies (9.8, 11.0, 12.0, 8.0, vs. 11.0) (10,14,15,16). In addition, despite the fact that there were no significant differences in median DEPS-R scores 11.0 (0-55) between females and males in these studies, in our sample, DEPS-R scores of males were slightly higher than in previous studies (9.3, 7.7, 9.4, 5.0, vs. 10.5).

Previous studies defined a score of DEPS-R ≥20 as an indicator of high risk for DEB (10,15,16). Twenty-five percent of our participants scored DEPS-R ≥20. Of these 67% were females and 33% were males. Thirty-one percent of the patients who scored ≥20 were not receiving enough insulin to cover the food when they overate, and 23% skipped the following insulin dose after overeating. While a greater proportion of the females scored above cut-off, surprisingly, more males reported insulin restriction and insulin omission in this group. Among females, insulin restriction and insulin omission rates were 28 and 22 % and among males these rates were 37.5 and 25 %, respectively. When insulin is omitted, with the catabolism of lipids andthe induced glycosuria results in excretion of calories with urine and contributes to weight loss (17,18). Insulin omission is associated with frequent events of diabetic ketoacidosis, and DEB is linked with recurrent episodes of severe hyperglycemia and about one-third of individuals with T1D intentionally omit insulin (8,19). Considering the social pressure about thinness and/or to be fit, young people with T1D are particularly at risk of weight loss practices such as insulin restriction or omission. Most studies focus on females but some researchers suggest that adolescent males with T1D also may have an increased risk of development of DEB (4). In our sample, as stated above, especially males appeared to be at risk for omission of insulin and insulin restriction. The use of a diabetes-specific screening instrument such as DEPS-R may be important for detection of the risk in both genders with T1D. Weight issue and external appearance may be the main problems in females but depressed mood, low self-esteem maybe a problem in boys. In order to further understand the increase in omission and restriction rate of insulin as well as other problems encountered in boys, these participants are under further psychiatric evaluation.

Consistent with the literature, in our series also, patients with scores above the cut-off on DEPS-R had significantly higher HbA1c levels (p=0.002) in both genders (10,15,20). No significant differences between females and males in relation to HbA1c was found. Participants with higher scores on the DEPS-R had a higher BMI-SDS, a finding which is in accordance with previous studies (10,14,15,20). In our study, median score of DEPS-R increased significantly from 7 in the underweight group to 10 in the normal weight group and to 22 in the obese group (p=0.012). Participants who were overweight (p≤0.0001) or obese (p≤0.0001) had higher scores on DEPS-R than those who were of normal weight. Females scored higher scores in all groups, but there was no significant difference between females and males. Olmsted et al (21) performed a longitudinal study on a sample of 126 T1D females age ranging from 9-13 years and reported that a higher BMI value predicted the onset of DEB. Wisting et al (14) in 770 children and adolescent with T1D (9-11 years old) reported that the prevalence of DEB increased considerably with increasing weight, especially for females. Markowitz et al (16), in their study on a sample of 43 patients of ages 10-17 years who were on insulin pump therapy, reported that participants who were overweight or obese scored higher on DEPS-R than those who were of normal weight. Thus, more weight and shape concerns, more negative feelings about one’s physical appearance, and more unhealthy weight control behaviors like insulin omission and insulin restricting can explain the relationship between BMI and DEB. As an exploratory analysis, we examined the stability of the DEPS-R scores by age groups. We found that median DEPS-R scores were 1.5 times and significantly higher in 90 adolescents aged 14-18 years than in 110 adolescents aged 9-13 years (p≤0.0001). Our results are similar to those reported in a previous DEPS-R validation study and a Norwegian sample study (10,14).

First DEPS-R validation study showed positive correlations of DEPS-R scores with age, age- and gender-adjusted BMI and HbA_1c_ levels (10).

Criterion validity of the Turkish version of the DEPS-R was shown through significant positive medium correlations with BMI-SDS (r=0.412, p<0.01) and HbA1c levels (r=0.303, p<0.01) and a small but positive correlation with age (r=0.194, p<0.01) that may have been influenced by the presence of DEB in children and adolescents with T1D. Therefore, in clinical settings, we recommend assessment of DEPS-R score in relation to BMI-SDS and age in T1D patients. Higher BMI-SDS, higher HbA1c, and older age appear to be risk factors for the development of DEBs among children and adolescents with T1D.

### Study Limitations

A major limitation of this study is its inability to validate the Turkish version of DEPS-R with a structured clinical diagnostic interview by a pediatric psychiatrist. This may have led to overestimation of DEB.

## CONCLUSION

Our data has shown that children and adolescents with T1D who have higher BMI values and higher HbA1c levels appear to exhibit more DEBs. DEB and insulin restriction were associated with poor metabolic control. Diabetes health care professionals should be aware of comorbid eating disorders in children and adolescent with T1D. A short, self-administered diabetes-specific screening tool for DEB can be used routinely in the clinical care of children and adolescents with T1D. The Turkish version of DEPS-R is a valid screening tool for DEB in T1D and it is potentially important to detect DEB at an early stage. We propose that future research should focus on the validity of the DEPS-R as compared with a structured clinical diagnostic interview.

## Figures and Tables

**Table 1:**
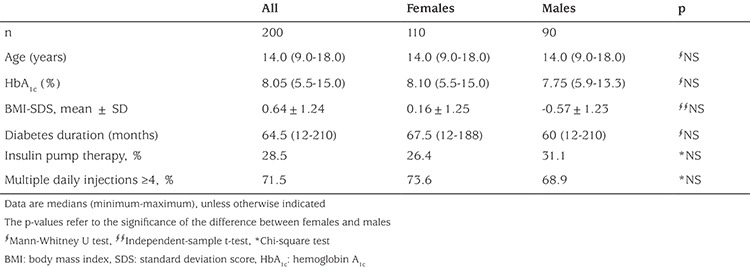
Characteristics of the study participant

**Table 2:**
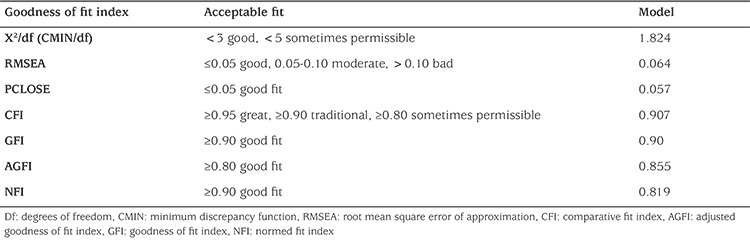
Results of confirmatory factor analysis

**Table 3:**
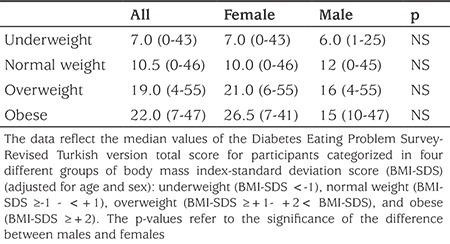
Median scores of Diabetes Eating Problem Survey-Revised according to categories of body mass index-standard deviation score

**Table 4:**
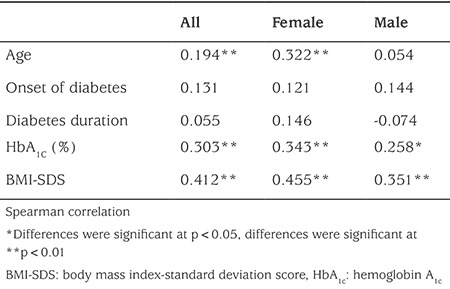
Correlations of Diabetes Eating Problem Survey-Revised Turkish version scores with age, body mass index-standard deviation score, hemoglobin A1c, and diabetes duration
